# Whole-Genome Sequence of a Multidrug-Resistant *Mycobacterium tuberculosis* Beijing Sequence Type 10 Isolate from an Outbreak in Thailand

**DOI:** 10.1128/genomeA.00803-14

**Published:** 2014-08-14

**Authors:** Olabisi O. Coker, Sanjib M. Regmi, Prapat Suriyaphol, Kwanrutai Chininmanu, Therdsak Prammananan, Angkana Chaiprasert

**Affiliations:** a Department of Microbiology, Faculty of Medicine, Siriraj Hospital, Mahidol University, Bangkok, Thailand; b Bioinformatics and Data Management for Research Unit, Office for Research and Development, Faculty of Medicine, Siriraj Hospital, Mahidol University, Bangkok, Thailand; c National Center for Genetic Engineering and Biotechnology, Pathumthani, Thailand

## Abstract

Infections with the Beijing family of *Mycobacterium tuberculosis* occur worldwide and are endemic in Asian countries. We present the draft genome sequence of DS6701, a multidrug-resistant *M. tuberculosis* Beijing strain of sequence type 10. The isolate is a representative of strains isolated from a multidrug-resistant tuberculosis outbreak in Thailand.

## GENOME ANNOUNCEMENT

The prevalence of drug-resistant and multidrug-resistant tuberculosis has a negative impact on the global control of tuberculosis (1). The Beijing family of *Mycobacterium  tuberculosis* has been shown to be associated with resistance to the most potent drugs against tuberculosis (2). Based on single nucleotide polymorphisms (SNPs), the Beijing family was grouped into different sequence types (3). Modern sublineage sequence type 10 is the most prevalent in countries such as Peru, Vietnam, and Thailand (4–6), and it is speculated to possess higher transmissibility and virulence (7). An outbreak of tuberculosis occurred in Thamaka district, Kanchanaburi province Thailand between 2002 and 2010 (8). Isolates from the outbreak were resistant to isoniazid, rifampin, ethambutol, and streptomycin. We performed the whole-genome sequencing of a representative strain, DS6701, isolated from a pulmonary tuberculosis patient during the outbreak.

Genomic DNA was purified by the hexadecyltrimethylammonium bromide (CTAB) method and sequenced on the Illumina Hiseq 2000 platform, yielding an average read length of 100 bp; 49,537,612 paired-end reads were generated and were aligned to reference the *M. tuberculosis* H37Rv genome (GenBank accession no. AL123456.2) with Bowtie 2 (version 2.1.0); 98.97% of the reads aligned to the reference genome. Analysis of the reads by BEDTools (version 17.2.0) showed that 99.3% of the reference genome was covered by at least one read.

The reads were trimmed and assembled by CLC Genomics Workbench (version 7.0; http://www.clcbio.com). A 4,343,026-bp draft assembly of 127 contigs was produced. The contigs have an average length of 34,180 bp, *N*_50_ of 98,718, and an average coverage of 1233×. The GC content of the genome is 65.6%. Annotation was performed by NCBI Prokarytoic Genome Annotation Pipeline (PGAP) (http://www.ncbi.nlm.nih.gov/genome/annotation_prok/). The draft genome has 3,998 genes, 3,918 coding sequences, 45 tRNAs, 3 rRNAs, 27 pseudogenes, and 5 noncoding RNAs.

Spoligotyping of the strain using Spolpred (9) showed a typical Beijing-specific spoligotype pattern that corresponds to Spoligotype International Type 1. SNP analysis was performed with SNPsfinder (http://snpsﬁnder.lanl.gov/). The DS6701 genome has 1,560 SNPs compared to the reference H37Rv genome. Compared to the genomes of other Beijing strains in GenBank, DS6701 has 1343, 763, 652, 432, 395, and 395 SNPs with the genomes of strains NITR203 (CP005082.1), R1207 (CM001045.1), CCDC5180 (CP001642.1), CCDC5079 (CP002884.1), X122 (CM001044.1), and HN878 (CM001043.1), respectively. SNP analysis according to (3) showed that it belongs to SNP cluster group 2 and modern sublineage ST 10 of the Beijing family.

A total of 229 SNPs comprising 72 synonymous, 104 nonsynonymous, and 53 intergenic were found to be unique to our strain. SNPs known to confer resistance to rifampin, (S450L in *rpoB* gene) isoniazid (S315T in *katG* gene), streptomycin (K43R in *rpsL* gene), and ethambutol (G406D in *embB* gene) were observed and confirmed the drug-resistant phenotypes.

The availability of the draft genome of DS6701 might provide a platform for comparative genomic analysis among modern sublineage sequence types of Beijing family isolates.

### Nucleotide sequence accession numbers.

This whole-genome shotgun project has been deposited at DDBJ/EMBL/GenBank under the accession number JOKR00000000. The version described in this paper is version JOKR01000000.
